# Can serum hyaluronic acid replace simple non-invasive indexes to predict liver fibrosis in HIV/Hepatitis C coinfected patients?

**DOI:** 10.1186/1471-2334-10-244

**Published:** 2010-08-19

**Authors:** Salvador Resino, José M Bellón, Cristina Asensio, Dariela Micheloud, Pilar Miralles, Ana Vargas, Pilar Catalán, Juan C López, Emilio Álvarez, Jaime Cosin, Raquel Lorente, María A Muñoz-Fernández, Juan Berenguer

**Affiliations:** 1 Laboratory of Molecular Epidemiology of Infectious Diseases, National Centre of Microbiology, Instituto de Salud Carlos III, Majadahonda, Madrid, Spain; 2 Biomedical Research Foundation, Hospital General Universitario "Gregorio Marañón", Madrid, Spain; 3 Agency for Health Technology Assessment, Instituto de Salud Carlos III, Madrid, Spain; 4 Internal Medicine Department, Hospital General Universitario "Gregorio Marañón", Madrid, Spain; 5 Infectious Diseases-HIV Unit, Hospital General Universitario "Gregorio Marañón", Madrid, Spain; 6 Microbiology Department, Hospital General Universitario "Gregorio Marañón", Madrid, Spain; 7 Pathology Department, Hospital General Universitario "Gregorio Marañón", Madrid, Spain; 8 Molecular Immunobiology Laboratory, Hospital General Universitario "Gregorio Marañón", Madrid, Spain

## Abstract

**Background:**

Hyaluronic acid (HA) serum levels correlate with the histological stages of liver fibrosis in hepatitis C virus (HCV) monoinfected patients, and HA alone has shown very good diagnostic accuracy as a non-invasive assessment of fibrosis and cirrhosis. The aim of this study was to evaluate serum HA levels as a simple non-invasive diagnostic test to predict hepatic fibrosis in HIV/HCV-coinfected patients and to compare its diagnostic performance with other previously published simple non-invasive indexes consisting of routine parameters (HGM-1, HGM-2, Forns, APRI, and FIB-4).

**Methods:**

We carried out a cross-sectional study on 201 patients who all underwent liver biopsies and had not previously received interferon therapy. Liver fibrosis was determined via METAVIR score. The diagnostic accuracy of HA was assessed by area under the receiver operating characteristic curves (AUROCs).

**Results:**

The distribution of liver fibrosis in our cohort was 58.2% with significant fibrosis (F≥2), 31.8% with advanced fibrosis (F≥3), and 11.4% with cirrhosis (F4). Values for the AUROC of HA levels corresponding to significant fibrosis (F≥2), advanced fibrosis (F≥3) and cirrhosis (F4) were 0.676, 0.772, and 0.863, respectively. The AUROC values for HA were similar to those for HGM-1, HGM-2, FIB-4, APRI, and Forns indexes. The best diagnostic accuracy of HA was found for the diagnosis of cirrhosis (F4): the value of HA at the low cut-off (1182 ng/mL) excluded cirrhosis (F4) with a negative predictive value of 99% and at the high cut-off (2400 ng/mL) confirmed cirrhosis (F4) with a positive predictive value of 55%. By utilizing these low and high cut-off points for cirrhosis, biopsies could have theoretically been avoided in 52.2% (111/201) of the patients.

**Conclusions:**

The diagnostic accuracy of serum HA levels increases gradually with the hepatic fibrosis stage. However, HA is better than other simple non-invasive indexes using parameters easily available in routine clinical practice only for the diagnosing of cirrhosis.

## Background

Human immunodeficiency virus (HIV) infection influences the natural evolution of chronic hepatitis C (CHC) infection by accelerating fibrosis progression and increasing the rate of cirrhosis and end-stage liver disease in HIV/hepatitis C virus (HCV) coinfected patients [1,2]. Despite a decline in morbidity/mortality from opportunistic infections since the introduction of highly active antiretroviral therapy (HAART), end-stage liver disease continues to be a frequent cause of hospitalization and death in patients coinfected with HIV and HCV [3,4].

Staging liver fibrosis is considered to be an essential part in the management of patients with CHC, because it provides prognostic information and, in many cases, assists in therapeutic decisions. For many years, the diagnosis and quantification of fibrosis have relied on liver biopsies, a procedure with several drawbacks (bleeding in the liver and around the site of the procedure, pain around the biopsy area, infection, damage to liver tissue, etc.) [5]. Currently, there are several non-invasive diagnostic methods for determining liver fibrosis that are being validated, such as blood markers and imaging methods [6].

One of these blood markers is hyaluronic acid (HA), an essential component of the extracellular matrix in virtually every tissue of the body. In the liver, HA is mostly synthesized by hepatic stellate cells and degraded by sinusoidal endothelial cells [7]. HA serum levels have been found to correlate with the histological stages of liver fibrosis in HCV monoinfected patients [8], and HA alone has shown very good diagnostic accuracy for the non-invasive assessment of fibrosis and cirrhosis [9,10].

The purpose of our study was to evaluate serum HA levels as a simple non-invasive diagnostic test to predict hepatic fibrosis in HIV/HCV-coinfected patients and to compare its diagnostic performance with other previously published simple non-invasive indexes consisting of routine parameters.

## Methods

### Patients

We carried out a retrospective cross-sectional study of all patients with documented HIV/HCV coinfection who underwent a liver biopsy between May 2000 and May 2007 at the HIV outpatient clinic of the Hospital Gregorio Marañón in Madrid (Spain).

Liver biopsies were performed on patients who were potential candidates for interferon plus ribavirin therapy and had no prior HCV therapy. The inclusion criteria were: availability of a frozen serum sample collected on the day of liver biopsy; no clinical evidence of hepatic decompensation; detectable HCV RNA by polymerase chain reaction; negative for hepatitis B surface antigen; CD4+ lymphocyte count higher than 200 cells/μL; antiretroviral therapy or no need for antiretroviral therapy; and absence of diabetes, active opportunistic infections, or active drug or alcohol addiction.

From our cohort of 361 patients with liver biopsy data, only 201 could be included because they had had a serum sample collected and frozen. The group of excluded patients had significant differences in the numbers of patients on antiretroviral therapy with NNRTI (included (104/201 patients (51.7%)) vs. excluded (57/160 patients (36.3%)), p < 0.05), advanced fibrosis diagnosis (included (41/201 patients (20.4%)) vs. excluded (18/160 patients (11.3%)), p < 0.05), undetectable plasma HIV viral load (included (156/201 patients (77.6%)) vs. excluded (90/160 patients (56.3%)), p < 0.05), plasma HCV RNA>850.000 copies/ml (included (125/201 patients (75.8%)) vs. excluded (95/160 patients (63.3%)), p < 0.05), and plasma AST levels (included (57 IU/L) vs. excluded (65.5 IU/L), p < 0.05).

All work was conducted in accordance with the Declaration of Helsinki. All patients gave their written informed consent for the liver biopsies, and the Institutional Ethics Committee approved the study.

### Clinical and Laboratory data

Immediately prior to the liver biopsy, a fasting blood sample was taken from the patient to analyze complete blood counts, liver panel, basic metabolic panel, coagulation tests, HIV viral load, and CD4^+ ^counts. The duration of HCV infection for all patients with a history of intravenous drug use was estimated from the first year needles were shared, and the others patients were considered to have an "unknown" HCV infection duration. Also, a serum sample was immediately frozen (-70°C) and stored for further assays.

HIV and HCV infection were documented in all patients by enzyme-linked immunosorbent assay (ELISA) and polymerase chain reaction (PCR). The HCV viral load was measured by PCR (Cobas Amplicor HCV Monitor Test, Branchburg, NJ, USA). HCV genotype was determined by hybridization of biotin-labeled PCR products to oligonucleotide probes bound to nitrocellulose membrane strips (INNO-LiPA HCV II, Innogenetics, Ghent, Belgium). HA was tested in serum samples by a commercially available quantitative ELISA (HA-ELISA; Echelon Biosciences Inc., Salt Lake City, UT, USA). Concentrations were assayed in duplicate.

For purposes of comparison with HA, we evaluated 5 reported simple models consisting of routine parameters to predict liver fibrosis: Forns [11], APRI [12], FIB-4 [13], HGM-1 and HGM-2 indexes [14] (Table 1).

**Table 1:** Simple non-invasive models for liver fibrosis consisting of routine parameters.

**Index**	**Mathematical formula**	**Reference**
		
**Forns**	7.811 - 3.131 * LN(Platelet count (10^9^/L) + 0.781 * LN(GGT) + 3.467 * LN (Age) -0.014*cholesterol	[11]
**APRI**	$\frac{100*(\frac{AST(IU/L)}{40})}{\text{Platelet count}(10^{9}/L)}$	[12]
**FIB-4**	$\frac{Age\;(years)*AST(IU/L)}{Platelet\;count(10^{9}/L)*\sqrt{ALT(IU/L)}}$	[13]
**HGM-1**	$\frac{1}{1+{\text{e}}^{\left(1.971+0.012*Platelet\;count(10^{9}/L)-0.026*\text{AST (IU/L)–}0.033*\text{glucose(mg/dL)}\right)}}$	[14]
**HGM-2**	$\frac{1}{1+{\text{e}}^{\left(6.175-0.010\;\text{ALP}\;(IU/L)-4.8*\text{INR}+0.010*Platelet\;count\;(10^{9}/L)-0.007*\text{AST}(IU/L)\right)}}$	[14]

Abreviations: LN, logarithm neperian; GGT, gamma glutamyl transpeptidase; AST, aspartate aminotransferase; ALT, alanine aminotransferase; INR, international normalized ratio; ALP, alkaline phosphatase.

### Liver biopsy and histology

Liver biopsies were performed on an outpatient basis following the recommendations of the Patient Care Committee of the American Gastroenterological Association [15]. All liver biopsies were performed by the same physicians (J.B. and P.M.) with a suction needle (HISTO-CUT 16G, Sterylab Srl. Milano, Italy). Ultrasound was routinely used to determine the percutaneous biopsy site. We did not systematically record the size of liver biopsy specimens, however, during the study period, only 5 out of 297 biopsies yielded insufficient liver tissue for pathological diagnosis.

The liver tissue sections were fixed in fomalin, embedded in paraffin and stained by hematoxylin-eosin, Mason's trichrome, and Perls' iron. The samples were evaluated by a single pathologist (E.A.). Liver fibrosis was estimated prospectively following the criteria established by the METAVIR Cooperative Study Group [16]. Fibrosis was scored as follows: F0, no fibrosis; F1, portal fibrosis; F2 periportal fibrosis or rare portal-portal septa; F3, fibrous septa with architectural distortion but with no obvious cirrhosis (bridging fibrosis); and F4, definite cirrhosis. The researchers in charge of evaluating the biopsies, interpreting the clinical data, or calculating and analyzing the reference standard had no prior knowledge of results.

### Statistical analysis

Overall, results are presented as medians (25^th ^percentile, 75^th ^percentile) for continuous variables and as frequencies and percentages for categorical data. Comparisons between HA levels and fibrosis stage were analyzed using the Mann-Whitney U-test. All tests were two-tailed with a p-value ≤ 0.05 considered to be significant. Statistical analysis was performed using SPSS 16.0 software (SPSS INC, Chicago, IL, USA) and STATA 9.1 (College Station, TX, USA).

We evaluated the diagnostic performance of all indexes using the receiver operating characteristic (ROC) curve constructed to study the absence and presence of significant fibrosis (F≥2), advanced fibrosis (F≥3), and cirrhosis (F4), and comparing the area under these ROC curves (AUC-ROCs) [17,18] with a nonparametric ROC analyses adjusted by Sidak's method for the effect of multiple comparisons.

For each fibrosis stage, we chose a low cut-off at 95% sensitivity (Se) used to predict the absence of the disease and a high cut-off at 95% specificity (Sp) used to predict the presence of the disease. Additional analyses of cut-offs that optimized both Se and Sp were also performed. The "optimal" cut-off was defined as the maximum of (Se + Sp). We calculated the Se, Sp, positive predictive value (PPV) and negative predictive value (NPV) for each cut-off point. We also calculated the diagnostic odds ratio (DOR) which expresses the strength of the association between test result and disease: it is the ratio of the odds of a positive result in a person with the target condition compared to a person without the condition [19]. A DOR of 1 suggests the test providing no diagnostic evidence. Moreover, we also calculated the likelihood ratios (LR) which describe how many times a person with the target condition is more likely to have a particular test result than a person without that condition. LRs contribute to change the probability that a target condition is present after the test has been made. Binary tests have two LRs, positive and negative (LR+, LR-). A LR of 1 indicates no diagnostic value.

Finally, we calculated the percentage of patients in whom the results of the HA could have avoided the biopsy. For this purpose, we constructed three new 2 × 2 contingency tables combining the two cut-off points for comparing the results that were lower than the low cut-off (discarding significant fibrosis) and the results that were higher than the high cut-off point (for significant fibrosis, for advanced fibrosis, and for cirrhosis, respectively) with the corresponding biopsy results. From each table, patients correctly classified (true positive (TP) + true negatives (TN)) by the test would not have needed the biopsy procedure.

## Results

### Patients

General characteristics of the 201 HIV/HCV-coinfected patients at the time of liver biopsy are shown in Table 2. Overall, 94.5% were on HAART: 23.4% with protease inhibitor based therapy, 51.7% with non-nucleoside analogue based therapy, 12.4% with 3 nucleoside analogue based therapy, and 7.5% with other drugs. The distribution of liver fibrosis in our cohort was 58.2% with significant fibrosis (F≥2), 31.8% with advanced fibrosis (F≥3), and 11.4% with cirrhosis (F4).

**Table 2:** Characteristics of the 201 HIV/HCV-coinfected patients, who underwent liver biopsies.

**Characteristic**	**Values**
	
No. HIV-1 patients *	201
Sex (male) *	152 (75.6)
**Age (years) **^**†**^	39.4 (36.8; 43.3)
HIV acquired by IVDU *	180 (89.6)
Prior AIDS *	62 (30.8)
**Years since HCV infection **^**†, ‡**^	21.3 (17.7; 24.3)
High alcohol intake ^*, §^	28 (14)
**Antiretroviral therapy**	
Non treated *	10 (5)
PI-based *	47 (23.4)
NNRTI-based *	104 (51.7)
3 NRTI-based *	25 (12.4)
Other *	15 (7.5)
**Months on HAART (n = 190) **^**†**^	50.2 (34.9; 65.7)
Stage of liver fibrosis *	
**F0**	16 (8)
**F1**	68 (33.8)
**F2**	53 (26.4)
**F3**	41 (20.4)
**F4**	23 (11.4)
**Fibrosis progression index **^**†**^	0.08 (0.05; 0.15)
**HIV markers**	
**Nadir CD4+ T-cells **^**†**^	210 (103; 324)
**Baseline CD4+ T-cells/μL **^**†**^	490 (373; 660)
HIV-RNA < 50 cp/mL *	156 (77.6)
**Log10 VL copies/mL (n = 45)**	3.23 (2.71; 3.98)
HCV markers *	
**HCV genotype**	
**1 or 4**	153 (77.3)
**3**	45 (22.7)
**HCV-RNA >850,000 cp/ml**	125 (75.8)
**Hematologic parameters **^**†**^	
**Platelet count (× 10**^**9**^**/L)**	177 (140; 221)
**Fibrinogen (mg/dL)**	259 (228; 305)
**INR**	1 (1; 1.02)
**Biochemical parameters **^**†**^	
**ALP (IU/L)**	124 (81; 196)
**AST (IU/L)**	57 (37.5; 85)
**GGT (IU/L)**	113 (58; 208)
**ALT (IU/L)**	77 (49; 117)
**AST/ALT**	0.75 (0.6; 0.97)

*Absolute number (percentage). ^†^Median (25^th ^percentile; 75^th ^percentile). ‡The duration of HCV infection for patients with a history of intravenous drug use (IVDU) was calculated starting from the first year needles were shared. Duration of HCV infection was considered to be unknown for subjects infected through sexual contact. §Patients were questioned in relation to alcohol consumption. The consumption of > 50 gr. of alcohol per day for ≥12 months was considered as a high intake.

Abbreviations: HCV, Hepatitis C virus; HIV-1, Human immunodeficiency virus type 1; IVDU, intravenous drug users; HAART, highly active antiretroviral therapy; NRTI, nucleoside analogue HIV reverse transcriptase inhibitor; NNRTI, non-nucleoside analogue HIV reverse transcriptase inhibitor; PI, protease inhibitor; HIV-RNA, HIV plasma viral load; HCV-RNA, HCV plasma viral load; INR, international normalized ratio; ALP, alkaline phosphatase; AST, aspartate aminotransferase; GGT, gamma glutamyl transpeptidase; ALT, alanine aminotransferase.

### Diagnostic performance

**Figure 1:**
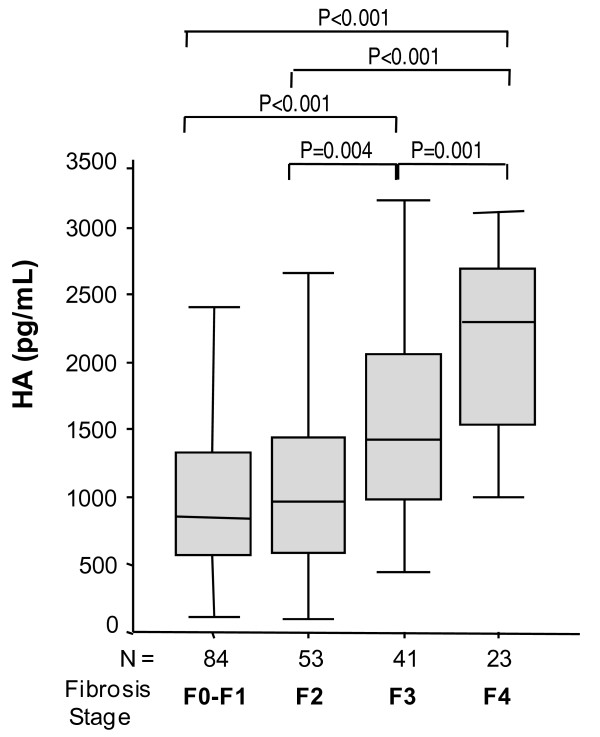
**Box plots illustrating the distribution of HA values against METAVIR fibrosis score**. Horizontal lines inside each box represent the median, and the lower and upper borders of the box encompass the interquartile range. The vertical lines from the ends of each box encompass the extreme data points.

**Figure 2:**
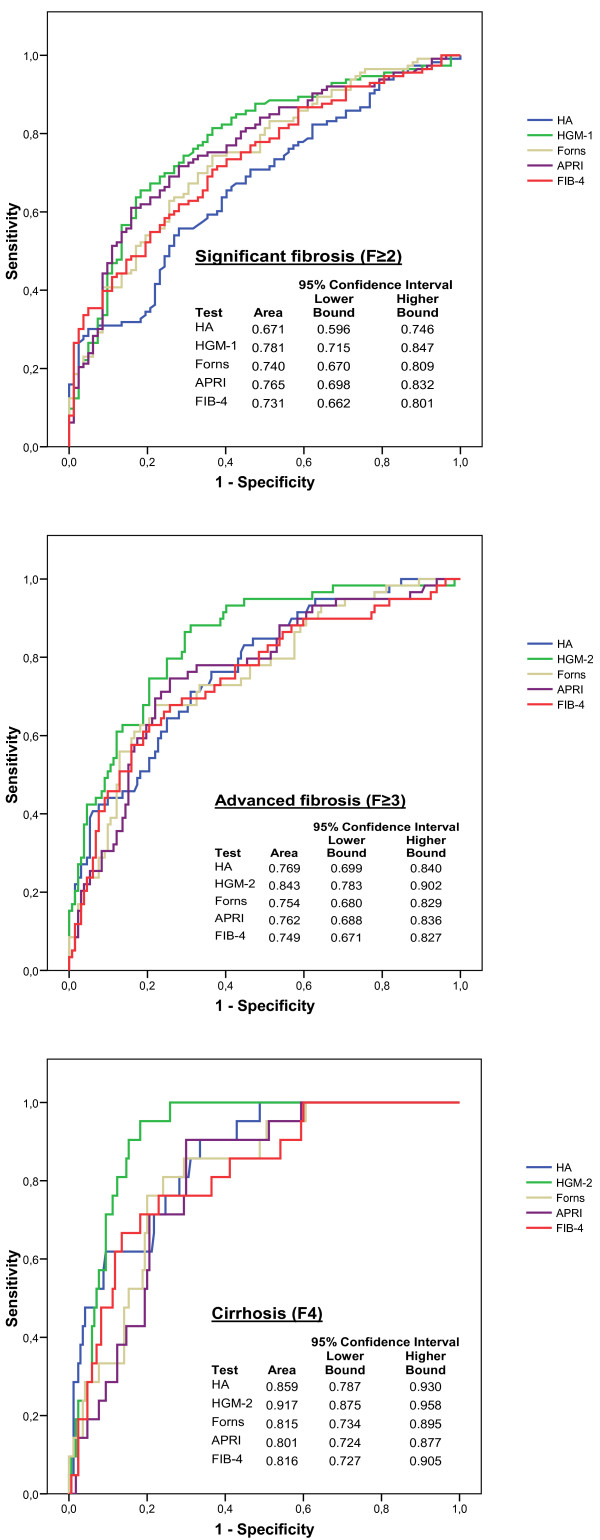
**Area under the receiver operating characteristic (AUC-ROCs) curves of HA, HGM-1, HGM-2, FIB-4, APRI and Forns indexes for significant fibrosis (F≥2), advanced fibrosis (F≥3) and cirrhosis (F4)**.

HA levels increased significantly with the stage of hepatic fibrosis (Figure 1A). The highest values of HA were found in cirrhotic patients. The AUC-ROC values of the HA for significant fibrosis (F≥2), advanced fibrosis (F≥3) and cirrhosis (F4) were similar to those of the Forns, APRI, FIB-4, HGM-1 and HGM-2 indexes (Figure 2B).

### Evaluation of cut-off points

For significant fibrosis (F≥2), using a low cut-off of 430 ng/mL HA, 13 of 84 (15.5%) patients with F<2 were correctly identified, and only 6 of 117 (5.1%) patients with F≥2 were misclassified (61.0% PPV and 68.4% NPV). With a high cut-off of 1800 ng/mL, 36 of 117 (30.8%) patients with F≥2 were correctly identified, and only 4 of 84 (4.8%) of patients with F<2 were misclassified (90% PPV and 49.7% NPV) (Table 3). Considering these low and high cut-off points, biopsies could have been avoided in 24.4% (49/201) of patients by using HA instead. When we applied an optimal cut-off of 1250 ng/mL, 128 patients were correctly identified (67 patients were TP and 61 patients were TN), and 83 patients were misclassified (23 patients were false positive (FP) and 50 patients were false negatives (FN)) (Table 3). The diagnostic accuracy estimates, the PPV, NPV, LR-, LR+ and DOR, all of them were not good enough.

**Table 3:** Diagnostic accuracy of hyaluronic acid (HA) measurement for significant fibrosis (F≥2), advanced fibrosis (F≥3) and cirrhosis (F4) in our cohort.

**Cut-off**	**TP**	**FP**	**TN**	**FN**	^(*) ^**Se** **(95% CI)**	^(*) ^**Sp** **(95% CI)**	^(*) ^**PPV** **(95% CI)**	^(*) ^**NPV** **(95% CI)**	**LR+** **(95% CI)**	**LR-** **(95% CI)**	**DOR** **(95% CI)**
											
**Significant fibrosis (F≥ 2)**											
							
**430 (ng/mL)**	111	71	13	6	94.9 (90.4 - 99.3)	15.5 (7.1 - 23.8)	61.0 (53.6 - 68.7)	68.4 (44.9 - 91.9)	1.12 (1.01 - 1.24)	0.33 (0.14 - 0.78)	3.39 (1.23 - 9.32)
**1250 (ng/mL)**	67	23	61	50	57.3 (47.9 - 66.7)	72.6 (62.5 - 82.7)	74.4 (64.9 - 84.0)	55.0 (45.2 - 64.7)	2.09 (1.43 - 3.06)	0.59 (0.46 - 0.75)	3.55 (1.9 - 6.5)
**1800 (ng/mL)**	36	4	80	81	30.8 (21.9 - 39.6)	95.2 (90.1 - 100)	90.0 (79.5 -100)	49.7 (41.7 - 57.7)	6.46 (2.39 - 17.46)	0.73 (0.63 - 0.84)	8.89 (3.02 - 26.13)
**Advanced fibrosis (F≥ 3)**											
							
**687 (ng/mL)**	61	88	49	3	95.3 (89.3 - 100)	35.8 (27.4 - 44.2)	40.9 (32.7 - 49.2)	94.2 (86.9 - 100)	1.48 (1.29 - 1.70)	0.13 (0.04 - 0.40)	11.32 (3.37 - 37.99)
**1250 (ng/mL)**	47	43	94	17	73.4 (61.8 - 85.0)	68.6 (60.5 - 76.7)	52.2 (41.3 - 63.1)	84.7 (77.5 - 91.8)	2.34 (1.75 - 3.12)	0.39 (0.25 - 0.6)	6.04 (3.1 - 11.7)
**2290 (ng/mL)**	18	7	130	46	28.1 (16.3 - 39.9)	94.9 (90.8 - 98.9)	72.0 (52.4 - 91.6)	73.9 (67.1 - 80.6)	5.50 (2.42 - 12.51)	0.76 (0.63 - 0.91)	7.27 (2.85 - 18.52)
**Cirrhosis (F4)**											
							
**1182 (ng/mL)**	22	78	100	1	95.7 (85.1 - 100)	56.2 (48.6 - 63.7)	22.0 (13.4 - 30.6)	99.0 (96.6 - 100)	2.18 (1.81 - 2.63)	0.08 (0.01 - 0.53)	28.21 (3.72 - 213.86)
**1320 (ng/mL)**	21	63	115	2	91.3 (77.6 - 100)	64.6 (57.3 - 71.9)	25.0 (17.1 - 34.9)	98.3 (95.5 - 100)	2.58 (2.04 - 3.26)	0.13 (0.04 - 0.51)	19.17 (4.4 - 84.4)
**2400 (ng/mL)**	11	9	169	12	47.8 (25.2 - 70.4)	94.9 (91.4 - 98.4)	55.0 (30.7 - 79.3)	93.4 (89.5 - 97.3)	9.46 (4.40 - 20.36)	0.55 (0.36 - 0.82)	17.21 (5.98 - 49.57)

For each fibrosis stage, we chose a low cut-off at 95% sensitivity (Se) used to predict the absence of the disease and a high cut-off at 95% specificity (Sp) used to predict the presence of the disease.

Abbreviations: TP, true positive cases (correct diagnosis); FP, false positive cases (over-diagnosis); TN, true negative cases (correct diagnosis); FN, false negative cases (missed cases); Se, sensitivity; Sp, specificity; PPV, positive predictive value; NPV, negative predictive value; LR, likelihood ratio; DOR, diagnostic odds ratio; (*): values as percentage (%).

For advanced fibrosis (F≥3), using a low cut-off of 687 ng/mL, 49 of 137 (35.8%) patients with F<3 were correctly identified, and only 3 of 64 (4.8%) patients with F≥3 were misclassified (40.9% PPV and 94.2% NPV). With a high cut-off of 2290 ng/mL, 18 of 64 (28.1%) patients with F≥3 were correctly identified and only 7 of 137 (5.1%) patients with F<3 were misclassified (72% PPV and 73.9% NPV) (Table 3). Considering these low and high cut-off points, biopsies could have been avoided in 33.3% (67/201) of patients by using HA instead. When we applied an optimal cut-off of 1250 ng/mL, 141 patients were correctly identified (47 patients were TP and 94 patients were TN), and 60 patients were misclassified (43 patients were FP and 17 patients were FN) (Table 3). The NPV value almost reached 85% but the PPV, LR-, LR+ and DOR were not good enough.

For cirrhosis (F4), using a low cut-off of 1182 ng/mL, 100 of 178 (56.2%) patients with F<4 were correctly identified, and only 1 of 23 (4.3%) patients with F4 was misclassified (22.2% PPV and 99% NPV). With a high cut-off of 2400 ng/mL, 11 of 23 (47.8%) patients with F4 were correctly identified, and only 9 of 178 (5.1%) patients with F<4 were misclassified (55% PPV and 93.4% NPV) (Table 3). Considering these low and high cut-off points, biopsies could have been avoided in 52.2% (111/201) of patients by using HA instead. When we applied an optimal cut-off of 1320 ng/mL, 136 patients were correctly identified (21 patients were TP and 115 patients were TN), and 65 patients were misclassified (63 patients were FP and 2 patients were FN) (Table 3). Using HA to predict cirrhosis, the NPV, LR-, and DOR value had good enough values.

## Discussion

In this study, we found that serum HA levels were positively correlated with the stages of liver fibrosis. Moreover, the AUC-ROC increased with the stage of fibrosis with the highest value found for cirrhosis. HA was moderately accurate at the diagnosis of F≥2 (AUC-ROC of 0.676), while it seemed to be a very useful method for the detection of cirrhosis (AUC-ROC of 0.863). Many fibrosis experts would consider non-invasive tests for fibrosis with an AUC-ROC value of 0.85-0.90 to be as good as liver biopsies for staging fibrosis [20]. Some authors have argued that some non-invasive markers of fibrosis might be even more accurate than biopsies and that most of the significantly discordant results between biopsies and non-invasive tests may be due to the method of obtaining biopsies that does not demonstrate the actual liver fibrosis state (sampling error when performing the biopsies) [21].

HA has been described as a component of several fibrosis indexes or as a single parameter for the non-invasive assessment of fibrosis/cirrhosis in HIV/HCV-coinfected patients [22-25]. In HIV/HCV-coinfected patients, there are few published studies with HA alone, and they are limited by their small sample sizes [25] or were designed to only evaluate significant fibrosis [23].

The diagnostic performance of HA was similar to the Forns, APRI, FIB-4, HGM-1 and HGM-2 indexes for our HIV/HCV-coinfected patients. We also found that the AUC-ROC of HA was similar to the AUC-ROC values of APRI, FIB-4, and Forns indexes obtained by other authors in HIV/HCV-coinfected patients [26-30] but lower than the AUC-ROC values of APRI, FIB-4, and Forns found in several studies carried out on HCV-monoinfected patients [11,12,31]. In summary, according to this study the performance of HA is not better than several biomarkers using parameters easily available in routine clinical practice in HIV/HCV-coinfected patients.

The clinical utility of HA in our study was low except for cirrhosis as the AUC-ROC for cirrhosis was the only one that was higher than 0.850. Also, the NPV was 99%, which could be acceptable for excluding cirrhosis. However, the PPV was only 55%, which is unacceptable for the diagnosis of cirrhosis; although this value can be explained due to the low number of cirrhotic patients in our cohort. Naturally, ruling out cirrhosis is of less importance in the management of patients than confirming such a diagnosis. According to all the available data, the practical interest of the isolated use of HA for assessing liver fibrosis in HIV/HCV-coinfected patients in clinical practice seems to be rather low.

The advantage of HA over the other simple non-invasive indexes (APRI, FIB-4, HGM-1 and HGM-2) is that these indexes could be affected by some factors associated with HIV infection such as biochemical and haematological abnormalities and antiretroviral therapy [32-34], which can lead to an increase in transaminases or cholesterol in the blood [32-34]. HAART has increased the incidence of significant metabolic disturbances. These metabolic disturbances produce clinical manifestations which have an impact on the future health of the HIV-infected patient, including hyperlipidaemia, lipodystrophy, metabolic syndrome, cardiovascular disease and type 2 diabetes [35,36]. Moreover, hepatotoxicity is a serious complication in patients taking HAART and coinfection with HCV increases the risk of liver toxicity while taking antiretroviral therapy [32]. HCV coinfection is associated with a 2 to 10-fold chance of developing elevated transaminase levels during HAART [33]. The evidence of severe hepatic dysfunction (coagulopathy or elevation of ammonia levels) is suggestive of severe toxicity and HAART should be discontinued. However, the simple indexes (APRI, FIB-4, HGM-1 and HGM-2) are calculated in a relatively easy way using parameters easily available in routine clinical practice. Even though HA is a single molecule, its quantification is not commonly measured in hospitals, it cannot be obtained from normal clinical data, and it is more expensive.

While HA has been shown to be accurate when used in combination with other parameters in HIV/HCV-coinfected patients (SHASTA index [24], HGM-3 [22]), its effectiveness at assessing liver fibrosis as an isolated marker is poorer. For instance, according to data published by our group very recently [22], the AUROC of HGM-3, a combination which includes HA, was 0.939 for F≥3, whereas the corresponding figure for HA alone reported here was 0.772. However, others authors have reported AUC-ROC values for Hepascore, Fibrometer and SHASTA (three indexes which include HA), and Fibrotest [30] similar to AUC-ROC values of HA in our patients.

Moreover, we used a commercial HA-ELISA test (Echelon Biosciences) different from the enzyme-linked protein binding assay (Hyaluronic Acid Test Kit, Corgenix, Westminster, CO, USA) or the sandwich enzyme binding assay kit (Pharmacia, Uppsala, Sweden) used by others authors [23,25,37-41]. To the best of our knowledge, this test has not previously been reported as a fibrosis test. So, this paper is a validation study of the HA-ELISA test (Echelon Biosciences) although the HA levels in this study are quite different (about 10 times larger) from those previously reported in HCV or HIV/HCV patients [23,25,38-41].

Aside from these laboratory biomarkers, liver fibrosis is evaluated using transient elastography (FibroScan) [42]. Our group reported an excellent diagnostic performance of liver stiffness for fibrosis and cirrhosis in HIV/HCV-coinfected patients [43], which was higher than the diagnostic performance of HA shown here.

The diagnostic performance analysis in our cohort had several limitations: a) the low number of patients; b) this study was made on patients with well preserved immune function and the extrapolation to individuals with more marked immune suppression would require further study; c) we did not directly compare HA with SHASTA, Fibrotest, Hepascore or Fibrometer because we did not have all the clinical routine variables needed to calculate these indexes the day the liver biopsy was undertaken, and as a result of the use of the HA-ELISA test (Echelon Biosciences) the values of these combining scores would be quite different from those previously reported; d) we could not give exact information regarding biopsy length or portal tracts, but we found that only 1.68% of biopsies were defective for pathological diagnosis, and these cases were excluded from this study; e) only one pathologist read the biopsies and the biopsies were not validated by someone else; f) the uneven distribution of the stages of fibrosis in our cohort with a high proportion of absent to mild fibrosis and a low proportion of cirrhosis (11%). However, we carried out an analysis using the DANA method, which is used when the distribution of fibrosis stages are highly asymmetric [44], and we did not find a significant increase in AUC-ROC values (*data not shown*).

## Conclusion

The diagnostic accuracy of serum HA levels increases gradually with the hepatic fibrosis stage. However, HA is better than other simple non-invasive indexes using parameters easily available in routine clinical practice only for the diagnosing of cirrhosis.

## Abbreviations

AUC-ROCS: Area under these ROC curves; CHC: Chronic hepatitis C; F≥2: Significant fibrosis; F≥3: Advanced fibrosis; F4: Cirrhosis; HA: Hyaluronic acid; HAART: Highly active antiretroviral therapy; HCV: Hepatitis C virus; HIV: Human immunodeficiency virus; NPV: Negative predictive value; PPV: Positive predictive value; SE: Sensitivity; SP: Specificity; LR: Likelihood ratio; DOR: Diagnostic odds ratio; TP: True positive cases (correct diagnosis); TN: True negative cases (correct diagnosis); FP: False positive cases (over-diagnosis); FN: False negative cases (missed cases).

## Competing interests

The authors declare that they have no competing interests.

## Authors' contributions

SR had primary responsibility for protocol development, participated in the design of the study performed the statistical analysis, and contributed to the writing of the manuscript. JMB, CA and AV participated in the design of the study, performed the statistical analysis, and contributed to the writing of the manuscript. RL carried out the immunoassays. EA had primary responsibility of fibrosis liver diagnosis. MMF, PM, PC, JCL, JC, and DM carried out patient screening, collecting and recording data, and contributed to the writing of the manuscript. JB conceived of the study, and participated in its design and coordination. All authors read and approved the final manuscript.

## Pre-publication history

The pre-publication history for this paper can be accessed here:

http://www.biomedcentral.com/1471-2334/10/244/prepub
